# Mycotoxins and Related Fungi in Italian Paddy Rice During the Growing Season and Storage

**DOI:** 10.3390/toxins11030151

**Published:** 2019-03-06

**Authors:** Terenzio Bertuzzi, Marco Romani, Silvia Rastelli, Paola Giorni

**Affiliations:** 1 Department of Animal, Food and Nutrition Science—DIANA, Università Cattolica del Sacro Cuore, Via Emilia Parmense 84, 29122 Piacenza, Italy; silvia.rastelli@unicatt.it; 2 Ente Nazionale Risi Rice Research Centre—Castello d’Agogna, 27030 Pavia, Italy; m.romani@enterisi.it; 3 Department of Sustainable Crop Production—DIPROVES, Università Cattolica del Sacro Cuore, Via Emilia Parmense 84, 29122 Piacenza, Italy; paola.giorni@unicatt.it

**Keywords:** rice, mycotoxins, growing season, storage

## Abstract

Mycotoxigenic fungi and relative mycotoxins contamination were monitored in Italian paddy rice samples both in field during the growing season and the first five months of storage. Three experimental fields, nine rice varieties and three sowing densities were considered; then, different lots of paddy rice were stored in warehouses at different temperature regimes. *Fusarium* spp. and *Aspergillus* spp. were found to be the fungi most likely to produce mycotoxins throughout the growing season. In particular, *A. flavus* and *A. niger* were found only rarely both in field and in post-harvest, while *A. versicolor* was always present although in low concentrations. *Penicillium* spp. strains were isolated sporadically and were found to be irrelevant in Italian rice fungal contamination. Sterigmatocystin (STC) was the main mycotoxin found in Italian rice, while aflatoxin (AFB_1_), deoxynivalenol (DON) and ochratoxin A (OTA) were rarely detected. Contamination generally increased from post-flowering to ripening; considering rice varieties, significant differences (*p* ≤ 0.01) were found in fungal contamination and STC production; no differences were observed between sowing densities. During storage, an increase in STC content was observed in higher temperature regimes, while all the other considered mycotoxins remained unchanged. These results indicated that contamination by STC, an emerging mycotoxin not legislatively regulated by the European Union, can be relevant in rice.

## 1. Introduction

Italy is the main rice producer in Europe, accounting for about 50% of the entire European production. Rice cultivation is mostly located in Northern Italy (Piedmont, Lombardy and Veneto), where water is relatively abundant and the rice crop can be cultivated in flooded fields; in 2018, the area dedicated to rice growing covered about 217,000 hectares ha and rice production amounted to nearly 1,451,319 tons [1]. Italian rice is used for different products; in particular, rice destined for baby foods requires minimal levels of undesired substances.

Rice, like other cereals, can be subject to contamination by mycotoxins, both in field and during storage. Several studies have reported mycotoxigenic fungi and mycotoxin occurrence in rice; in particular *Aspergillus*, *Fusarium* and *Penicillium* Genera [2,3,4] with their possible related aflatoxins (AFs), ochratoxin A (OTA) and deoxynivalenol (DON) have often been investigated [5,6,7,8,9]. The maximum limits for these mycotoxins in rice were set by the European Commission [10,11]. Recently, sterigmatocystin (STC), an emerging toxin considered by the International Agency for Research on Cancer (IARC) as a class 2B compound (possible human carcinogen), has often been detected in paddy rice and derived products [12,13]. However, little data has been reported on mycotoxigenic fungi occurrence and mycotoxin contamination in rice cultivated in Italy; this makes it impossible to evaluate the possible risk of contamination or to declare that if it is more relevant during the growing season in field or during storage. Unlike for maize and wheat, little information has been reported about the agronomic practices, meteorological conditions and storage methodologies that might influence and reduce mycotoxin contamination in rice.

The aim of this study was to monitor the occurrence of mycotoxigenic fungi and mycotoxins in Italian paddy rice samples during the growing season and the storage period in order to verify possible influences of rice variety, sowing density and storage techniques.

## 2. Results and Discussion

### 2.1. Mycotoxigenic Fungi Contamination in the Field

The sampling time was shown to play an important role in fungal presence in field. In particular, fungi were found to occur most frequently at ripening with a significant decrease if given an extra 15 days in field (Table 1). This was probably due to environmental conditions less prone to fungal development, in particular a lower a_w_ level of rice kernels. Only *A.* section *Flavi* and *A. versicolor* showed a different behavior, the former being highest at flowering and the latter in over-ripening (Table 1). However, the incidence of *A.* section *Flavi* remained always lower than 1%, indicating that this is not a problem for Italian rice. This is in contrast with studies conducted in other areas; in tropical countries, for example, *A. flavus* seems to be the main fungus for rice grains with high contaminations both in field and in post-harvest [2,7]. This is probably due to the high temperatures and high relative humidity recorded throughout the year in these countries, conditions that are quite different from the areas where rice is cultivated in Italy. Differently from *A. flavus*, *A. versicolor* had a low but more stable presence during the growing season, resulting to be more able to adapt even when a_w_ level decreased. It is well known that this fungus is generally able to grow at low a_w_ levels being xerophilic [14,15]. This was also demonstrated in a previous study on fungi found in cheese where *A. versicolor* resulted to have an optimal a_w_ for growth lower than other fungal species [16].

Fungi belonging to the genus *Fusarium* were found to be present at all the sampling times considered with the only exception of flowering; their presence slowly increased up to harvest time but without showing significant differences (Table 1). Usually, different mycotoxigenic *Fusarium* species are reported in rice such as *F. graminearum*, *F. fujikuroi* and *F. proliferatum* [17]. In our study, the presence of *Fusarium* species was found to be 20% of the total fungal contamination, which is the highest level of all the mycotoxigenic fungi considered. This is in accordance with another study conducted in Brazil where *Fusarium* species were found to be the most common of the fungi isolated from rice grains at harvest [18].

Considering rice varieties, significant differences (*p* ≤ 0.01) were found in fungal contamination; in particular, CL26, CLXL 745, Mare CL, Centauro, Sole CL and Sirio CL were found to be more prone to fungal infection (≥45%) while Terra CL was the least contaminated (31.5%) (Table 1). Significant differences between rice varieties were also noted in mycotoxigenic fungi infection with Centauro and CL15 more contaminated by *Fusarium* spp., CL 26 more contaminated by *A. versicolor* and Sirio CL highly contaminated by both *Fusarium* and *A. versicolor*; no significant differences between rice varieties were noted in *A.* section *Flavi* infection (Table 1). In rice, as in other cultivations, variety can play an important role in fungal presence and, consequently, in mycotoxin contamination. Differences between rice varieties regarding fungal contamination had already been highlighted in a previous study [19]; further, an important role can be played by the starch content of the variety, as found for other cereals like maize [20]. Rice varieties with a high content of starch seem to be more resistant, in particular to aflatoxin accumulation [21].

Species of the genus *Penicillium* have rarely been isolated and can be considered uninfluential for both plant infection and mycotoxins production in field. *Penicillium* species, in fact, are usually reported in rice only during the post-harvest period [3].

Interestingly, no significant differences in fungal population were observed either between long and round rice varieties (data not shown) or between the different sowing densities used in field (Table 1). No data were found in literature regarding previous studies investigating the possible effect of rice grain typology or sowing densities on fungal incidence.

### 2.2. Mycotoxin Occurrence in Field

**Figure 1:**
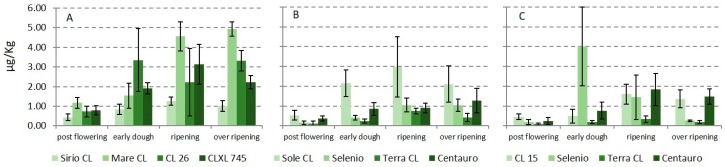
Occurrence of sterigmatocystin in paddy rice samples collected during growing season in three different experimental fields: (**A**) long rice varieties; (**B**,**C**) round rice varieties.

The contamination of rice samples collected from the experimental fields during the growing season varied greatly. STC was the most widespread mycotoxin, detected in all paddy rice samples and at all sampling times with the only exception of flowering when it was not detected (Figure 1).

STC increased from post-flowering to ripening, remaining constant or slightly decreasing in samples collected 15 days after ripening. The contamination by STC ranged from 0.16 to 8.34 µg kg^−1^; rice variety influenced the level of contamination. At ripening, mean STC contamination was lower than 1 µg kg^−1^ in 4 rice samples (all round grain varieties), while it was higher than 2 µg kg^−1^ in other 4 (3 long B grain and 1 round grain varieties)., Mare CL (long B grain) and Sole CL (round grain) variety showed a significant higher contamination respect to other varieties, both at ripening and at post-ripening (*p* = 0.01). High values were also found in CL26 and CL XL 745 varieties. Contamination of the three common varieties cultivated in two fields showed slightly different results. Terra CL and Centauro varieties showed low and similar STC contamination in both experimental fields, while Selenio had not always consistent results in considered fields; a justification of this trend could be the heterogeneity of the STC contamination. As for fungal contamination, no significant difference was observed between the sowing densities. The occurrence of STC in rice has already been reported; in particular, recent surveys [12,13] showed a widespread STC contamination in paddy rice and derived products. STC has generally been considered a storage mycotoxin [22]; however, the data reported in this study showed that STC already occurred in field at not negligible levels and that the contamination can be strongly influenced by the rice varieties. In 2013, the European Food Safety Authority (EFSA) published a scientific opinion stating that STC in grains and grain-based products could lead to an exposure in the range 1.5–8 µg/kg that can be considered of low health concern. However, no exposure data were available and a risk characterization was not possible for this mycotoxin [23]. A more recent review published in 2018, reporting recent toxicological studies, showed that STC should be taken into account for its ability to have a significant impact on human and animal health [24].

**Figure 2:**
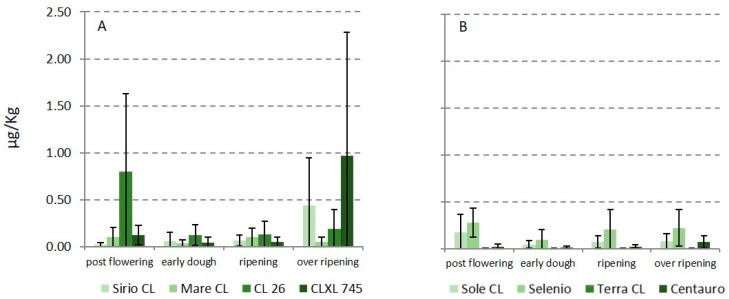
Occurrence of aflatoxin B1 in paddy rice samples collected during the growing season in two different experimental fields: (**A**) long B grain varieties; (**B**) round grain varieties.

In terms of AFs, aflatoxin B_1_ (AFB_1_) was detected in few samples (Figure 2), mainly at ripening (highest level was 2.46 µg kg^−1^), always lower than the limit of 5 µg kg^−1^ fixed by the European Union (EU) for rice and maize subjected to sorting or other physical treatment before human consumption [10]; further, results for the replicates varied greatly, showing a high standard deviation and a not-uniform contamination.

Contamination with AFs in Italian paddy rice is generally absent or present in traces; probably, high temperature and drought, which occurred in summer 2017, increased the risk of their contamination.

Contamination with DON was very low. Even if Genera *Fusarium* was often isolated in rice samples, DON was detected at low levels in only 18 samples (the highest contamination was 64 µg kg^−1^ in post-ripening). These data confirmed the low risk of contamination reported by previous surveys. In Brazil, a total of 91% of rice samples (*n* = 165) did not contain detectable DON [25], while only 1 of 5 samples collected in China was contaminated (139 µg kg^−1^) [26]. Two surveys carried out in Korea showed a low incidence of contamination [27,28]. Regarding OTA, no sample showed a contamination higher than the limit of detection (0.02 µg kg^−1^). Several studies reported not negligible contaminations with OTA in rice [6,29,30,31]; however, there was little data that referred to rice produced in Italy in the literature.

### 2.3. Fungal Contamination during Storage

Fungal presence both as total fungi and as different mycotoxigenic species did not show significant differences during the storage period (Table 2) remaining similar to fungal composition obtained in field. This demonstrated that the period in field is the most crucial for rice fungal contamination when good management in post-harvest is possible. In particular, it is well known that for species belonging to Genera *Fusarium* and *Aspergillus* invasion starts in field but is only in the event of poor post-harvest practices that it can worsen [4]. Interestingly, differences between the 2 different storage techniques were found only in total fungi incidence, which resulted higher in the case of RT storage, as expected. Temperature is effectively one of the most important factors able to influence fungal development during storage [32,33].

For Carnaroli, the only rice variety stored both at RT and HT and for this reason analysed separately from the others, significant differences were found only in total fungi incidence (47% vs 30%) while the two kinds of refrigeration can be considered similar for all mycotoxigenic fungal species considered. Even in this case, temperature resulted crucial for fungal containment as already reported also for other cereals like maize [34].

### 2.4. Mycotoxin Contamination during Storage

**Figure 3:**
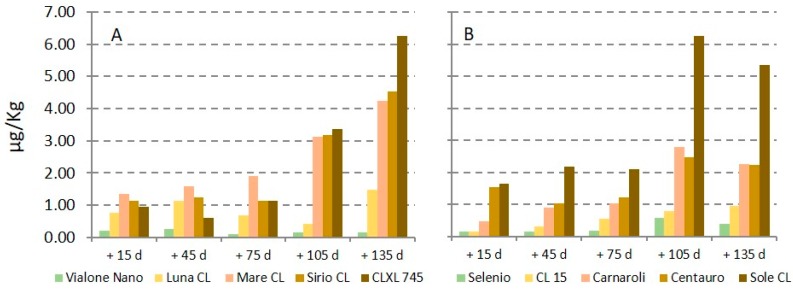
Mean sterigmatocystin (STC) contamination (µg/kg) during storage at medium refrigeration (**A**) and at room temperature (**B**).

For rice lots coming from in field trials and successively stored, both RT and MR conditions showed an increase in STC contamination during storage; the data obtained at the end of storage were significantly higher than those at the beginning (*p* ≤ 0.01) (Table 2). This trend was also confirmed by the results of other lots; in particular, STC contamination increased when already present at not negligible level at the beginning of the storage period (Figure 3).

From an initial level between 0.94 and 1.64 µg kg^−1^, STC exceeded 3 µg kg^−1^ during storage (maximum value 6.25 µg kg^−1^) while no increase was observed for lots showing an initial low STC level. As regards the paddy rice lots of the Carnaroli rice variety stored both at HR and at RT conditions, different trends were observed. No increase of STC contamination was observed during HR storage, showing values between 0.19 and 0.65 µg kg^−1^. Contrarily, contamination increased from 0.45 to 2.79 µg kg^−1^ during RT storage.

**Figure 4:**
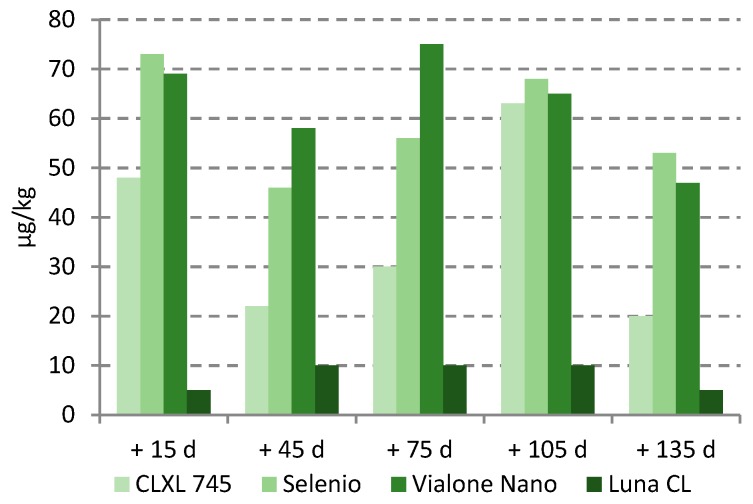
Mean deoxynivalenol (DON) contamination (µg/kg) during storage.

AFB_1_ was detected in only four paddy rice lots, stored at RT or MR condition, at levels always lower than 0.6 µg kg^−1^ and contamination did not show an increase during the storage period. The same trend was observed for DON; in three paddy rice lots low contamination was found (from 19 to 75 µg kg^−1^) and with no increase during storage (Figure 4). Finally, OTA was never found, as well as in samples collected from the field.

## 3. Conclusions

Italian paddy rice was considered for mycotoxin risk and important information was found for both in field and storage period. *Fusarium spp*. and *A. versicolor* were found to be to be the most important fungi for mycotoxins in Italian rice, being present from flowering to post-harvest on grains. STC was almost always found in field samples, demonstrating that it should not be considered only a storage mycotoxin; it was detected in all samples after flowering and during storage, while AFB_1_, DON and OTA rarely occurred. Contamination generally increased from post-flowering to ripening and variety seemed to play an important role in fungal presence and mycotoxin contamination. On the contrary, sowing density did not show any significant difference. However, these data should be confirmed by evaluating the contamination in subsequent growing seasons. In storage, STC was the main mycotoxin and the only one able to increase in both MR and RT conditions.

These results showed how mycotoxin contamination in Italian rice can be considered not relevant with the only exception of STC, an emerging mycotoxin neither routinely determined, nor legislatively regulated by the EU.

## 4. Materials and Methods

### 4.1. Field Samples

Rice samples were collected at different rice growing stages (flowering (BBCH 69), 15 days post-flowering (BBCH 77), early dough (BBCH 83), full ripening (BBCH 89) and over ripening (BBCH 92, 15 days post-ripening) in three experimental fields located near Mortara (PV) in Lombardy, the main Italian rice production region. Nine rice varieties, both long and round grain (Table 3), and three sowing densities (100, 150 and 200 kg/ha of seeds) were considered. For each experimental field, four varieties were cultivated; three rice varieties were common for two experimental fields. Soil texture and sowing period varied, while meteorological conditions can be considered similar because of the proximity of the fields (all within 10 km). For each rice variety and field trial, three replicates (500 g each) were collected and analyzed separately. Grain samples were collected directly from the experimental field. Each rice variety was cultivated on an area of about 600 m^2^; this area was divided in three subareas of 200 m^2^ representing replicates. For each subarea, plants were collected following an X-shape design and grains obtained by plant shelling were considered as representative for the subarea. A total of 60 samples of 500 g were collected from each experimental field along the growing season (1.5 Kg for each rice variety x each sampling time). Moreover, two other sowing densities were tested on similar areas and following the same approach (1.5 Kg for each rice variety x each sowing density x each sampling time) for a total of 120 samples. The whole experiment considered 180 rice samples of 1.5 Kg each for a total of 270 kg of rice grains. Samples were used for mycological analyses and then dried, milled using a cyclone hammer mill (1-mm sieve, Pulverisette, Fritsch GmbH, Idar-Oberstein, Germany), homogenized and kept at 4 °C until chemical analysis.

### 4.2. Storage

In Italy, rice is generally stored at room temperature (RT); however, the technique based on medium refrigeration (MR) has recently become widespread. Storage at high refrigeration (HR) is sometimes used for rice destined for baby-food. For this reason, lots of four paddy rice varieties cultivated in the field (about 10 tons for each) were harvested, fast dried to 12% of moisture and then stored for five months (from November 2017 to March 2018) in two different conditions:

- Medium refrigeration (MR) at 11–15 °C in a conventional warehouse (2 rice varieties: Sirio CL and Mare CL)
- Room temperature (RT) in a conventional warehouse (2 rice varieties: Sole CL and Centauro)

Further, another six paddy rice lots (about 30 tons each), not used in the *in-field* trial, were stored in different conditions in order to evaluate differences in storage management. In particular, 3 rice varieties (Vialone Nano, Luna CL and CL XL 745) were stored at MR regime, another 3 rice varieties (Carnaroli, Selenio and CL 15) at RT regime. In addition, an aliquot of Carnaroli variety (about 15 tons) was stored in steel silos at 1–2 °C (High refrigeration, HR).

The first sampling during storage was scheduled 15 days after harvest time in order to achieve the expected temperature for MR and HR storage; then, samples were collected every 30 days at different points of the silo/warehouse using a sample probe to obtain a global sample of about 10 kg. Five sampling times and three replicates of each rice variety were considered. The samples were used for mycological analyses and then dried, milled using a cyclone hammer mill (1-mm sieve), homogenized and a final sample of 1 kg kept at 4 °C until chemical analysis.

### 4.3. Fungal Identification

Rice panicles were hand shelled and 50 kernels were randomly collected from each sample and replicate, surface disinfected and transferred on Petri dishes containing potato dextrose agar (PDA, Biolife, Milano, Italy). After incubation at 25 °C for 5–7 days (12 h light photoperiod), the incidence of kernels infected by fungi was quantified (total fungi).

The identification of *Fusarium* spp. and *Penicillium* spp. isolates at Genus level was based on observations with binocular microscope (×40); only for *Aspergillus* spp. isolates, identification was carried out to species level according to Raper et al. [35].

### 4.4. Reagents and Standards

The chemicals and solvents used for the extraction and clean-up solutions were ACS grade or equivalent (Carlo Erba, Milan, Italy); deionized water was purified through a Milli-Q treatment system (Millipore, Bedford, MA, USA). For LC-MS/MS analysis, water, methanol, acetonitrile and formic acid were HPLC grade (Merck, Darmstadt, Germany). Phosphate saline buffer (PBS) was prepared as follows: NaCl 8 g L^−1^, KCl 0.2 g L^−1^, Na_2_HPO_4_ 1.15 g L^−1^, KH_2_PO_4_ 0.2 g L^−1^; pH 7.4. AFs, OTA and DON standard solutions were prepared as previously described [36,37,38].

The STC analytical standard was obtained from Sigma-Aldrich (Milan, Italy), while the internal standard [^13^C_18_]-sterigmatocystin (96.4% ^13^C) was purchased from Biopure (Tulln, Austria) as standard solution in acetonitrile (1.2 mL, 25.7 μg mL^−1^, uncertainty 1.02 μg mL^−1^). Stock and working STC standard solutions were prepared and calibrated spectrophotometrically as reported by Bertuzzi et al. [16].

### 4.5. Analysis for Mycotoxin Determination

The analyses were carried out using methods developed in our department; in particular, AFs were determined by HPLC-FLD as reported by Bertuzzi et al. [36]; OTA by HPLC-FLD [37], DON by GC-MS [38], STC by LC-MS/MS [13]. Briefly, AFs were extracted from 25 g of sample with 250 mL of acetone-water 7 + 3 *v*/*v* using a rotary-shaking stirrer for 60 min. After purification through an immunoaffinity column, the extract was filtered (Millex HV 0.45 mm) before HPLC-FLD analysis (Jasco Corporation, Tokyo, Japan); the average recovery for AFB_1_ was 95.8 ± 3.4% (three replicates at two spiking levels, 2.0 and 10.0 µg kg^−1^). The limit of detection (LOD) and quantification (LOQ), defined at those levels resulting in signal-to-noise ratios of 3 and 10, were 0.05 and 0.15 µg kg^−1^, respectively. OTA was extracted from a 10 g of sample with 100 mL of a mixture of 0.13 M sodium bicarbonate-methanol (5 + 5 *v*/*v*) for 45 min using a rotary-shaking stirrer. After purification through an immunoaffinity column, the eluate was concentrated under a gentle stream of nitrogen, brought to 2 mL with acetonitrile-water (41 + 59 *v*/*v*), vortex-mixed for few seconds and filtered before HPLC-FLD analysis. The mean recovery (mean of 3 replicates at 2 spiking levels) was 95.2 ± 3.4%; LOD and LOQ were 0.02 and 0.06 µg kg^−1^, respectively. DON was extracted from samples (25 g) with 100 mL of acetonitrile-water (86 + 14 *v*/*v*); an aliquot (6 mL) of the filtrate was slowly pressed through a MycoSep 227 column. An aliquot (200 µL) of the internal standard diacetoxyscirpenol (DAS 10 mg L^−1^) was added to 2 mL of the eluate. The solution was evaporated to dryness and derivatized with 200 µL of trimethilsilylimidazole-trimethilclorosilane (1 + 0.2 *v*/*v*) for 15 min in subdued light. Then 0.8 mL hexane was added, and the solution was washed with 1 mL 0.2 M phosphate buffer pH 7.5, and the hexane phase was used for GC-MS. GC-MS analysis was carried out using a TraceGQ Ultra coupled with an ISQ single quadrupole mass spectrometry (Thermo-Fisher Scientific, San Jose, CA, USA). The analysis was carried out using a capillary column Rtx-5MS, 30 m × 0.25 mm i.d., 0.25 µm film thickness. LOD and LOQ were 5 and 15 µg kg^−1^, respectively; the average recovery was 92.4 ± 2.6%. STC was extracted from an aliquot of 20 g taken from the milled sample with 100 mL acetonitrile-water 8 + 2 *v*/*v* using a rotary-shaking stirrer for 60 min. After purification through an immunoaffinity column, the extract was concentrated under a gentle flow of nitrogen and brought to 1 mL with acetonitrile-water 4 + 6 *v*/*v*. An aliquot of 900 µL of cleaned extract was transferred into an autosampler vial and mixed with 100 µL of isotopically labelled STC (12 µL L^−1^). A volume of 20 µL of the extract was injected into an LC-MS/MS system consisting of a LC 1.4 Surveyor pump, a Quantum Discovery Max triple-quadrupole mass spectrometer (Thermo-Fisher Scientific, San Jose, CA, USA) and a PAL 1.3.1 sampling system (CTC Analytics AG, Zwingen, Switzerland). STC was chromatographed on a Betasil RP-18 column (5 µm particle size, 150 × 2.1 mm, Thermo-Fisher) with a gradient acetonitrile-water (both acidified with 0.2% formic acid; flow rate 0.2 mL min^−1^); the ionization was performed using positive atmospheric pressure chemical ionization (APCI). The matrix effect was low, less than 10%; the LOD and the LOQ were 0.05 and 0.15 µg kg^−1^, respectively. The average recovery was 90.4% ± 4.2%.

### 4.6. Data Analysis

Data on fungal incidence were arcsine transformed, while mycotoxin content in flour was ln transformed before statistical analysis [39]. The statistical package IBM SPSS Statistics 21 (IBM Corp., Armonk, NY, USA) was used for the analysis of variance (ANOVA). Mean separation was done with the Tukey test (*p* ≥ 95%) to highlight significant differences.

## Figures and Tables

**Table 1:** Analysis of variance (ANOVA) of fungal incidence and contamination of sterigmatocystin (STC) and aflatoxin B_1_ (AFB_1_) at different sampling times in different rice varieties cultivated at three different sowing densities.

Factor	Total Fungi Incidence (%)		Incidence of *Fusarium* spp. (%)		Incidence of A. *versicolor* (%)		Incidence of *A. flavus* (%)		STC (µg/kg)		AFB_1_ (µg/kg)	
**Sampling time (A)**	**		**		**		**		**		**	
Flowering (BBCH 69)	0.3	e	0.0	d	0.0	c	0.0	b	0.0	d	0.0	b
15 days post-flowering (BBCH 77)	15.6	d	3.1	c	0.7	bc	0.8	a	0.4	c	0.1	ab
Early dough (BBCH 83)	26.5	c	2.2	c	1.6	ab	0.1	b	1.4	b	0.1	ab
Full Ripening (BBCH 89)	89.0	a	18.9	a	1.3	ab	0.1	b	2.0	a	0.1	ab
Over ripening (BBCH 92)	67.6	b	10.4	b	2.2	a	0.0	b	1.6	ab	0.2	a
**Rice variety (B)**	**		**		**		N.S.		**		**	
Centauro	45.6	a	11.7	ab	0.6	bc	0.2		1.0	bcd	0.0	b
Selenio	38.9	ab	7.8	bcd	0.3	c	0.2		1.1	cd	0.1	ab
Sole CL	45.2	a	4.8	cd	0.7	bc	0.0		1.9	a	0.1	ab
Terra CL	31.5	b	5.1	d	0.2	c	0.2		0.3	e	0.0	b
CL26	55.1	a	4.4	cd	4.1	a	0.0		1.9	ab	0.3	a
CLXL 745	54.3	a	7.3	abcd	1.3	b	0.5		1.6	abc	0.2	ab
Mare CL	52.5	a	4.4	cd	1.1	bc	0.5		2.4	a	0.1	ab
Sirio CL	49.5	a	12.5	a	4.8	a	0.3		0.7	de	0.1	ab
CL15	40.3	ab	10.5	abc	0.2	c	0.2		1.0	bcd	0.0	b
**Sowing density (C)**	N.S.		*		N.S.		N.S.		N.S.		N.S.	
100 kg/ha	65.8		8.2	ab	2.4		0.3		1.7		0.1	
150kg/ha	60.3		11.1	a	2.0		0.1		1.8		0.1	
200kg/ha	61.2		7.5	b	2.3		7.5		1.9		0.1	
**AxB**	**		**		**		**		**		N.S.	
**AxC**	N.S.		N.S.		N.S.		N.S.		N.S.		N.S.	
**BxC**	N.S.		*		**		*		N.S.		N.S.	
**AxBxC**	N.S.		N.S.		**		**		N.S.		N.S.	

Different letters mean significant differences according to Tukey Test; N.S.: not significative; *: *p* ≤ 0.05; **: *p* ≤ 0.01.

**Table 2:** Analysis of variance (ANOVA) of fungal incidence and contamination of sterigmatocystin (STC) at different sampling times with storage at room temperature (RT) and at medium refrigeration (MR).

Factor	Total Fungi Incidence (%)		Incidence of *Fusarium* spp. (%)		Incidence of A. *versicolor* (%)		Incidence of *A. flavus* (%)		STC (µg/kg)	
**Sampling Time (A)**	N.S.		N.S.		N.S.		N.S.		**	
+15 days	41.1		10.8		0.1		0.0		1.2	b
+45 days	51.0		7.7		1.2		0.1		1.3	b
+75 days	47.1		12.4		1.8		0.0		1.3	b
+105 days	50.3		11.7		0.4		0.0		2.4	ab
+135 days	48.5		3.6		0.3		0.0		3.3	a
**Storage (B)**	**		N.S.		N.S.		N.S.		N.S.	
MR	40.7	b	9.6		0.8		0.0		1.7	
RT	68.8	a	9.3		0.9		0.1		2.2	
**AxB**	N.S.		N.S.		N.S.		N.S.		N.S.	

Different letters mean significant differences according to Tukey Test; N.S.: not significant; *: *p* ≤ 0.05; **: *p* ≤ 0.01.

**Table 3:** Varieties and grain type of rice cultivated in the three experimental fields used for in field trial.

Rice Varieties	Field A	Grain Type	Field B	Grain Type	Field C	Grain Type
1	CLXL 745	LONG B	SOLE CL	ROUND	CL15	ROUND
2	CL26	LONG B	SELENIO	ROUND	SELENIO	ROUND
3	SIRIO CL	LONG B	CENTAURO	ROUND	CENTAURO	ROUND
4	MARE CL	LONG B	TERRA CL	ROUND	TERRA CL	ROUND
